# Amplification of very low output voltages of PV panels using a Duffing oscillator

**DOI:** 10.1016/j.heliyon.2024.e38982

**Published:** 2024-10-09

**Authors:** Hermann Frederic Emakoua, Guy Valery Ayissi Eyebe, Annie Sylvie Wakata, Jean Sire Armand Eyebe Fouda, Martin Kom

**Affiliations:** a Department of Physics, Faculty of Science, University of Yaounde I, P.O. Box: 812, Yaounde, Cameroon; b Institute of Mathematics, University of Kassel, Kassel, Germany; c Electrical Engineering of National Advanced School of Engineering of Yaounde, University of Yaounde I, P.O. Box: 8390, Yaounde, Cameroon

**Keywords:** Solar cell, Duffing oscillator, Nonlinear amplification

## Abstract

We are interested in the amplification of very low voltages produced by solar cells during sunset or weak sunshine. The study uses a device consisting of a Duffing oscillator, which amplifies and automatically regulates a low-voltage input, an inverter that reverses the negative voltage of one of the outputs of the oscillator, and a summing device to add the voltages of the two oscillator outputs. Experimental and theoretical investigations are conducted, and it is observed from the results that the output voltage can reach to 7.03V for an input voltage of 0.43V, i.e. a gain of 24.3dB. Furthermore, we observe from the amplification factor, and the behavior of the cyclic ratio that the whole system nonlinearly amplifies the input voltage with a large factor for weak inputs. We finally compare the results produced by the proposed system and those produced by a conventional boost converter at very low voltages. It then results that the Duffing oscillator provides large amplification factors for low voltages.

## 1 Introduction

Energy supply has become one of the major concerns of our world today. Every country is now looking for more sustainable solution that is cheap, easily developed and non-polluting energy sources such as solar and wind power. The development of technologies that can help achieving such a goal is crucial. Photovoltaic (PV) energy is a clean and renewable energy source, which offers advantages such as environmentally friendly and low-maintenance electricity production [1], [2]. However, the output voltage of solar cells strongly depends on the amount of sunlight and temperature [3], [4], [5], [6], [7]. In other words, as the solar irradiation decreases, the output of the PVs also decreases to very low voltage [8]. This concern has led research to focus on improving the efficiency of solar cells [9], [10]. Xin li & al. proposed in [11] mixed 2D/3D perovskite solar cells (PSC), a system capable of solving the efficiency, scalability and stability problems faced by certain solar cells. Some authors are more interested in optimizing the conversion inverter. A modified interleaved inverter was then proposed by Manesh Patel & al. in [12]. In the same direction, Xin Zhang & al. proposed in [13] an inverter capable of supporting very small voltages. Pelap & al. in [14] proposed a model that replaces the series resistor of the PV with a non-linear resistor. Such a model suggests an improvement of the efficiency by 1.41%. In [15], the authors are interested in reducing the leakage current, thus in improving the performance of solar cells. To find the maximum power point (MPP), Akhil Raj & R.P. Praveen [16] proposed a DC-DC Boost converter which, thanks to its “Advanced Pertub & Observation” (APO) MPP tracking (MPPT) algorithm, offers an efficiency greater than 95% compared to the traditional DC-DC Boost converter. Also with the aim of improving the efficiency of photovoltaic cells, several works based on the combination of PV and boost choppers were undertaken [2], [16], [17], [18]. In these choppers, the cyclic ratio is generally used to further increase the voltage boosting capacity [19], [20]. Considering for example a conventional DC-DC boost converter, the maximum value of the duty cycle does not generally exceed 0.9 which is equivalent to an amplification gain of about 20dB [19]. However, despite the work done to improve the amplification of low voltages from solar cells, even with the possibility of obtaining 10 times the input voltage via choppers, the efficiency remains low. The problem of improving solar cell technologies for efficient amplification of very low input voltages to acceptable output voltages still persists.

In this paper, we are interested on the non-linear amplification in order to improve the conversion efficiency. For this purpose, we propose a DC-DC boost converter with adaptive duty cycle, using a Duffing oscillator. The Duffing oscillator is one of the systems that allow the study of nonlinear dynamics [21], [22]. It corresponds to a second order non-linear differential equation with or without forcing [21], [22]. The response of the Duffing oscillator highly depends on the nature of the forcage [21], [22]. For a zero or constant input signal, a constant response amplitude is obtained as output [22], [23]. For an alternative or variable input signal, a variable response amplitude is obtained at the output, exhibiting some times chaotic behaviors well analyzed in the literature [23], [24], [25]. In the case of a constant input signal, the amplification ratio between the constant amplitude of the output signal and the constant amplitude of the input signal can be studied. In this situation, the analysis of the Duffing oscillator as amplifier, unknown to our knowledge in the literature can be done. Indeed, due to the nonlinear behavior of the Duffing oscillator a comparison between the amplifications through the Duffing oscillator and the well-known DC-DC boost converters is presented in order to show the high performance of the nonlinear effect on the amplification of two signals.

In order to present the performance of our proposed converter, the rest of the paper is organized as follows: in section 2, we present the system with the corresponding modeling equations; section 3 is devoted to the analysis of the system dynamics, the amplification factor and a comparative study between the proposed system and the conventional DC-DC Boost converter, as well as the experimental investigation of the proposed system to confirm the theoretical prediction; and a conclusion is drawn in section 4.

## 2 Materials and methods: physical system of the proposed device

### 2.1 Circuit modeling

**Figure 1:**
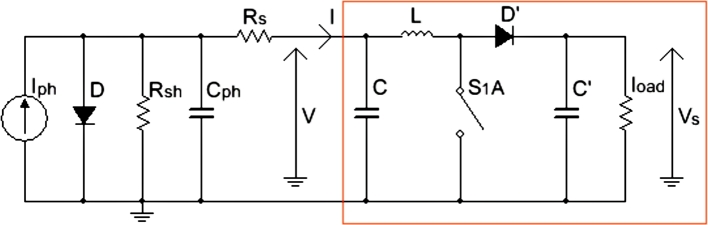
Electrical diagram of a solar cell with a DC-DC Boost Chopper (red box part of the figure).

The equivalent electrical circuit of the solar cell is depicted in Fig. 1. $I_{ph}$ represents the generated current under a given irradiance. *D* is a diode whereas, $R_{sh}$ and $R_{s}$ represent respectively the shunt and series resistors of the device. In the literature, the shunt resistance is very large and the series resistance is very small [26]. $C_{ph}$ is the parasitic capacitance responsible for the leakage current [27], [28].

The output voltage of this cell is generally low during sunset or weak sunshine. A DC-DC boost chopper was connected to its output [16], [17], [18]. This results in the PV with DC-DC Boost Chopper circuit diagram shown in Fig. 1. As previously mentioned, the disadvantage of the DC-DC boost chopper is its constant and limited ratio of output and input voltages, called amplification factor. This amplification ratio is related to the cyclic ratio *σ*, and the relationship between the chopper output voltage $V_{s}$, the chopper input voltage *V*, which is at the same time the PV output voltage, and this duty cycle is given by [20]:

(1) $$V_{s}=\frac{v}{1-\sigma }$$

**Figure 2:**
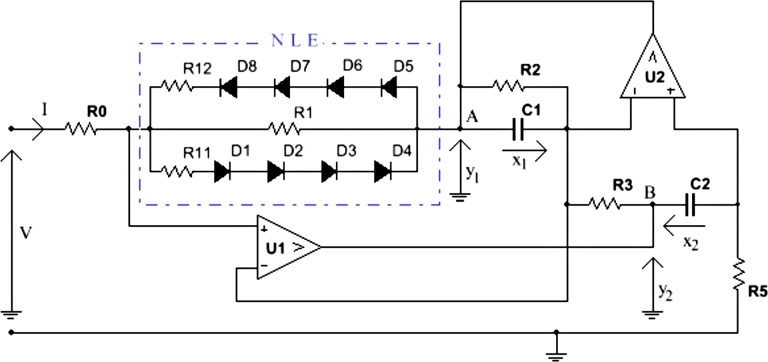
Electrical diagram of the Duffing oscillator.

**Figure 3:**
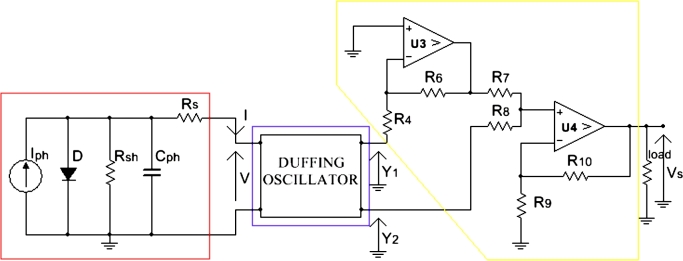
Electrical diagram of the complete device.

From Eq. (1), since both input and output voltages must be positive, an amplification of *V* is obtained when *σ* is between 0 and 1. The amplification ratio is maximized when σ→1. Furthermore, by increasing the cyclic ratio from 0 to 1 in order to raise this amplification ratio, for high voltages after the PV, the output voltage would be very high and would risk deteriorating the load. Hence, depending on the output voltage *V* of the cell, adapting the amplification ratio of each chopper requires varying its cyclic ratio each time. Consequently, an amplification and self-regulating system is needed. In our work, we replace the DC-DC boost chopper with a new block, the electrical Duffing oscillator, to handle this limitation. The diagram of the Duffing oscillator is given in Fig. 2 [29], [30] where $X_{1}$ and $X_{2}$ are voltages across capacitors $C_{1}$ and $C_{2}$, respectively.

$$y_{1}=-X_{1}+R_{5}C_{2}\frac{dX_{2}}{dt},$$

and

$$y_{2}=X_{2}+R_{5}C_{2}\frac{dX_{2}}{dt}$$

are the output voltages, collected at nodes A and B respectively, of the Duffing oscillator. Diodes $D_{1}$-$D_{8}$ are combined to generate the cubic nonlinearity that is necessary to model the Duffing oscillator equation known as

(2) $$\overset{¨}{x}+\delta \overset{˙}{x}+(\beta_{1}x^{3}\pm \omega_{0}x)=\gamma \cos(\omega t),$$

where $\overset{˙}{\left(\cdot \right)}=\frac{d(\cdot )}{dt}$, *δ* is the damping term, *γ* and *ω* are respectively the amplitude and the frequency of the forcing term. 4 diodes are cascaded in order to increase the diode cut-in voltage [29], [31]. It was shown in [32], [29], [30] that the current flowing through the non-linear element (NLE), composed with resistors $R_{1}$, $R_{11}$, $R_{12}$ and diodes $D_{1}$-$D_{8}$, is a polynomial function of the third order of the voltage at the NLE terminal. This current is expressed as follows:

(3) $$I=f(X_{1})=pX_{1}+qX_{1}^{3},$$

where $X_{1}$ is the voltage across capacitor $C_{1}$, *p* and *q* are characteristic parameters of the NLE experimentally determined. By replacing the Boost Chopper block in Fig. 1 with the Duffing block shown in Fig. 2, we obtain the schematic of the device under investigation in Fig. 3. Since the Duffing oscillator has two opposite outputs, i.e. one with a positive voltage and the other one with a negative voltage, an inverter and a summing device are added to the system. The inverter reverses the negative output of the oscillator, while the summing device adds both positive and reverse negative voltages.

### 2.2 Modeling equations

Starting from the solar cell in Fig. 1, and according to the Kirchhoff's laws [33], of nodes, photocurrent $I_{ph}$ can be decomposed as follows:

(4) $$I_{ph}=I_{d}+I_{R_{sh}}+I_{C_{ph}}+I,$$

where $I_{d}$ is the current through the diode *D*, $I_{R_{sh}}$ is the current through the shunt resistor $R_{sh}$, $I_{C_{ph}}$ is the current through the parasitic capacitor $C_{ph}$ and *I* the steady-state current out of the solar cell. The definitions of the currents $I_{d}$, $I_{R_{sh}}$ and $I_{C_{ph}}$ are respectively:

(5) $$I_{d}=I_{s}\left[\exp\left(\frac{v+R_{s}I}{nv_{T}}\right)-1\right]$$

(6) $$I_{R_{sh}}=\frac{v+R_{s}I}{R_{sh}}$$

(7) $$I_{C_{ph}}=C_{ph}\frac{d\left(v+R_{s}I\right)}{dt}$$

Since *I* is a steady-state current, its rate of change with time is zero, i.e. dI/dt=0. Therefore, Eq. (7) becomes:

(8) $$I_{C_{ph}}=C_{ph}\frac{dv}{dt}$$

According to Fig. 3, solar cell current *I* enters directly into the Duffing block, which feeds particularly the non-linear element in this block.

(9) $$C_{ph}\frac{dv}{dt}+C_{ph}R_{S}p\frac{dX_{1}}{dt}+C_{ph}R_{S}q\frac{dX_{1}^{3}}{dt}=I_{ph}-I_{s}\left[\exp\left(\frac{v+pR_{S}X_{1}+qR_{S}X_{1}^{3}}{nv_{T}}\right)-1\right]-\frac{v+pR_{S}X_{1}+qR_{S}X_{1}^{3}}{R_{sh}}-pX_{1}-qX_{1}^{3}$$

In order to see the contribution of the new block in the PV amplification, the voltage across it has to be determined. Applying Kirchhoff's laws to the Duffing circuit in Fig. 2 and considering the ideal operational amplifiers (OPAMP), we obtain the equation below [34]:

(10) $$\left\{ \begin{array}{c} \frac{dX_{1}}{dt}=\frac{1}{C_{1}R_{3}}X_{2}-\frac{1}{C_{1}R_{2}}X_{1}\\ \frac{dX_{2}}{dt}=\frac{1}{C_{2}R_{5}}V-\frac{R_{0}}{C_{2}R_{5}}f\left(X_{1}\right) \end{array}\right.$$

where $f\left(X_{1}\right)$ is given by Eq. (3). Eq. (10) can be rewritten in terms of a single voltage. By considering only the voltage $X_{1}$, it becomes:

(11) $$\frac{d^{2}X_{1}}{dt^{2}}+\frac{1}{C_{1}R_{2}}\frac{dX_{1}}{dt}+\frac{R_{0}p}{C_{1}C_{2}R_{3}R_{5}}X_{1}+\frac{R_{0}q}{C_{1}C_{2}R_{3}R_{5}}X_{1}^{3}=\frac{V}{C_{1}C_{2}R_{3}R_{5}}$$

By analyzing the summing-inverter block marked in yellow in Fig. 3 for R4=R5=R6=R7=R8=R9 and considering the ideal OPAMP of the block, we can express the output voltage $V_{s}$ of the whole device as follows [35]:

(12) $$V_{S}=\frac{R_{9}+R_{10}}{R_{7}+R_{8}}\left[\left(\frac{R_{6}R_{8}}{R_{4}R_{9}}+\frac{R_{3}}{R_{2}}\right)X_{1}+\frac{C_{1}R_{3}R_{7}}{R_{9}}\frac{dX_{1}}{dt}\right]$$

Eqs. (9), (11) and (12) thus define the overall electrical behavior of the system in Fig. 3. For a good numerical analysis, the dimensioning of these expressions is required. By taking

$X_{1}=R_{S}I_{S}y;\;V=R_{S}I_{S}x;\;V_{S}=R_{S}I_{S}z;\;t=R_{S}C_{0}\tau$

we obtain, dimensionless equation below [36]

(13) $$\left\{ \begin{array}{c} \overset{¨}{y}+a_{1}\overset{˙}{y}+a_{2}y+a_{3}y^{3}=a_{4}x\\ \overset{˙}{x}+b_{1}\overset{˙}{y}+3b_{2}y^{2}\overset{˙}{y}=b_{4}-b_{3}\{\exp\left[b_{5}\left(x+b_{1}y+b_{2}y^{3}\right)\right]\\ -1\}-b_{6}\left(x+b_{1}y+b_{2}y^{3}\right)-b_{1}b_{3}y-b_{2}b_{3}y^{3}\\ z=\alpha y+\beta \overset{˙}{y} \end{array}\right.$$

where, $a_{1}=\frac{R_{S}C_{0}}{R_{2}C_{1}};\;a_{2}=pR_{0}a_{4};\;a_{3}=qR_{0}{\left(R_{S}I_{S}\right)}^{2}a_{4};$

$a_{4}=\frac{{\left(R_{S}C_{0}\right)}^{2}}{R_{3}R_{5}C_{1}C_{2}};\;b_{1}=pR_{S};\;b_{2}=qR_{S}{\left(R_{S}I_{S}\right)}^{2};$

$b_{3}=\frac{C_{0}}{C_{ph}};\;b_{4}=\frac{I_{ph}}{I_{S}}b_{3};\;b_{5}=\frac{R_{S}I_{S}}{nV_{T}};\;V_{T}=\frac{k_{B}T}{e};\;b_{6}=\frac{R_{S}}{R_{sh}}b_{3}$

$\alpha =\frac{R_{9}+R_{10}}{R_{7}+R_{8}}\left(\frac{R_{6}R_{8}}{R_{4}R_{9}}+\frac{R_{3}}{R_{2}}\right);$

$\beta =\frac{R_{9}+R_{10}}{R_{7}+R_{8}}\left(\frac{R_{3}R_{7}C_{1}}{R_{9}R_{s}C_{0}}\right)$

## 3 Results and discussion

### 3.1 Dynamical behavior of the system 2

**Figure 4:**
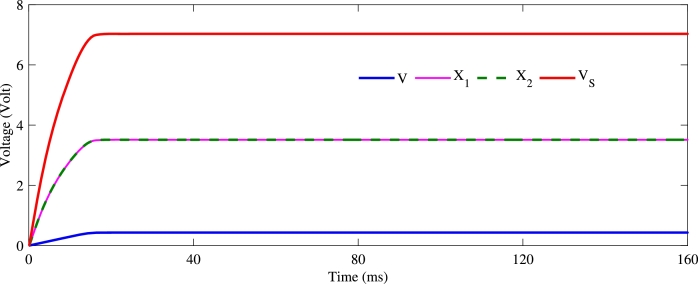
Evolutions of voltages *V*, *X*_1_, *X*_2_ and *V*_*s*_ as function of time.

The system is simulated using Fortran 95. Taking into account some simulation constraints such as the forcing frequency ω=0, instead of $\omega =2\pi f_{0}$ with $f_{0}=6.67kHz$ as defined in [29], some parameters of the Duffing circuit were modified to enhance the amplification of low voltages. The simulation parameters used in this section are tabulated in Table 1. Starting from Eq. (13) and considering the parameter setting in Table 1, we show in Fig. 4 the temporal evolution of the output voltage *V* of the solar cell, both voltages $X_{1}$ and $X_{2}$ from the Duffing oscillator, and the global output voltage $V_{s}$. From this figure, due to the considered initial conditions, a short transient regime is observed for all the different voltages. The most important remark is the global behaviors of these voltages for long times. They take constant values as the time change. This means that the system operates in DC mode as in agreement with the PV technology [37], [38]. Moreover, we notice that for an input voltage V=0.43V, the output voltage $V_{s}$ is 7.03*V*, i.e. an amplification factor of about 16.4 or a gain of 24.3 dB. Voltages $X_{1}$ and $X_{2}$ are taking the same value of 3.5*V*, thus corresponding to an amplification factor of 8.2 approximately.

**Table 1:** System parameters.

*R*_0_ = 1*k*Ω	*R*_8_ = 1*k*Ω	*C*_1_ = 47*nF*
*R*_1_ = 5.1*k*Ω	*R*_9_ = 1*k*Ω	*C*_2_ = 20*nF*
*R*_2_ = 1*k*Ω	*R*_10_ = 1*k*Ω	$C_{ph}=1000\;\mu \text{F}$
*R*_3_ = 1*k*Ω	*R*_11_ = 830Ω	*I*_*s*_ = 1.41*nA*
*R*_4_ = 1*k*Ω	*R*_12_ = 830Ω	*I*_*ph*_ = 30*mA*
*R*_5_ = 1*k*Ω	*R*_*s*_ = 0.16Ω	*k*_*B*_ = 1.38 × 10^−23^*J*/*K*
*R*_6_ = 1*k*Ω	*R*_*sh*_ = 700Ω	*e* = 1.6 × 10^−19^*C*
*R*_7_ = 1*k*Ω	$C_{0}=1\;\mu \text{F}$	*T* = 298.15*K*

### 3.2 Amplification factor and comparison between the proposed model and the DC-DC boost

#### 3.2.1 Determination of the amplification factor

In the case of the solar cell with DC-DC boost chopper system shown in Fig. 1, according to Eq. (1), the amplification ratio is expressed as [19]:

(14) $$\frac{V_{s}}{v}=\frac{1}{1-\sigma }$$

From equation (14), it appears that 1/(1−σ) represents the amplification factor of the chopper block. Referring to Fig. 4, we have seen that for subsequent times, after the transient regime, all voltages stabilize at constant values. This allows us to conclude that for high times (t≫0) all time derivatives cancel out. Therefore, Eq. (13) can be rewritten as follows:

(15) $$\left\{ \begin{array}{c} a_{2}y+a_{3}y^{3}-a_{4}x=0\\ b_{4}-b_{3}\{\exp\left[b_{5}\left(x+b_{1}y+b_{2}y^{3}\right)\right]\\ -1\}-b_{6}\left(x+b_{1}y+b_{2}y^{3}\right)-b_{1}b_{3}y-b_{2}b_{3}y^{3}=0\\ z=\alpha y \end{array}\right.$$

In the case of the linear system, i.e. $a_{3}=0$, the combination of the first and third equations in Eq. (15) reduces to:

(16) $$z=\frac{a_{4}}{a_{2}}\alpha x;$$

Hence, for $\frac{a_{4}}{a_{2}}\alpha >1$ the system amplifies the input voltage *x*. The term $\frac{a_{4}}{a_{2}}\alpha$ that we agree to call linear amplification coefficient is directly related to the linear coefficient *p* of the NLE as $\frac{a_{4}}{a_{2}}=\frac{1}{pR_{0}}$. We can therefore conclude that in the absence of the non-linearity, the proposed system behaves like a conventional chopper where the amplification factor is constant regardless of the value of the input voltage.

In the case of the non-linear system, given by Eq. (15), resolving the first equation by the Cardan method [39], we obtain the voltage *y* as a function of the input voltage *x*:

(17) $$y={\left(\frac{\frac{a_{4}x}{a_{3}}+\sqrt{{\left(\frac{a_{4}x}{a_{3}}\right)}^{2}+\frac{4a_{2}^{3}}{27a_{3}^{3}}}}{2}\right)}^{\frac{1}{3}}+{\left(\frac{\frac{a_{4}x}{a_{3}}-\sqrt{{\left(\frac{a_{4}x}{a_{3}}\right)}^{2}+\frac{4a_{2}^{3}}{27a_{3}^{3}}}}{2}\right)}^{\frac{1}{3}}$$

By replacing Eq. (17) in the last equation of Eq. (15) on the one hand and expressing the ratio z/x on the other hand, we obtain the amplification factor of the Duffing block given by Eq. (18):

(18) $$g\left(x\right)=\alpha {\left(\frac{a_{4}}{2a_{3}x^{2}}+\sqrt{{\left(\frac{a_{4}}{2a_{3}x^{2}}\right)}^{2}+\frac{a_{2}^{3}}{27a_{3}^{3}x^{6}}}\right)}^{\frac{1}{3}}+\alpha {\left(\frac{a_{4}}{2a_{3}x^{2}}-\sqrt{{\left(\frac{a_{4}}{2a_{3}x^{2}}\right)}^{2}+\frac{a_{2}^{3}}{27a_{3}^{3}x^{6}}}\right)}^{\frac{1}{3}},$$

where

(19) $$g\left(x\right)=\frac{z}{x}.$$

**Figure 5:**
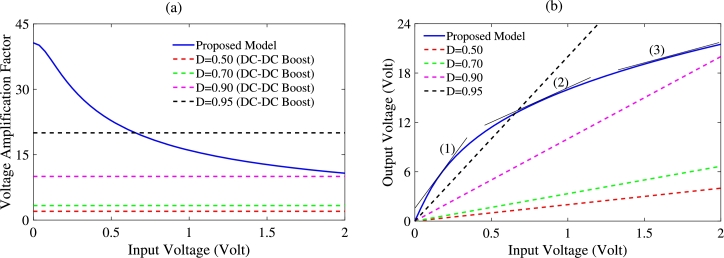
Amplification curve via the Duffing block. (a): Amplification factor *V*_*s*_/*V* as a function of *V*. (b): Output voltage *V*_*s*_ as a function of the input voltage *V*.

From this equation, we can see that the amplification factor is a nonlinear function of *x*, i.e. the dimensionless form of the input voltage of *V*. This means that the value of the amplification factor or the gain will change depending on the value of the input voltage. Thus, there is a kind of self-regulation / adaptation of the amplification factor, depending on the value of the input voltage. These results are confirmed by the blue curve in Fig. 5, showing the amplification factor of the Duffing block as a function of the input voltage of the block. From Fig. 5(a) we notice that for small values of the input voltage, this amplification is large and, as the value of the voltage from the PV becomes larger, the amplification decreases. This means that for low solar energy levels, the Duffing block adopts a very high amplification ratio as verified in Fig. 5(b). For example, for an input voltage of 0.1*V*, the output voltage is 3.8*V*, which corresponds to an amplification factor of 38 equivalent to a gain of 31.6 dB. For an input voltage of 1.2*V*, the theoretical amplification factor drops to 14.4, thus corresponds to an output voltage of 17.3*V*. This variation of the amplification factor according to the input voltage confirms the fact that the proposed model adapts the gain in order to optimally amplify output voltage of the Duffing oscillator block. Consequently, the proposed device constitutes an interesting energy harvester.

#### 3.2.2 Comparison between the DC-DC boost and the Duffing oscillator-based amplifier

**Figure 6:**
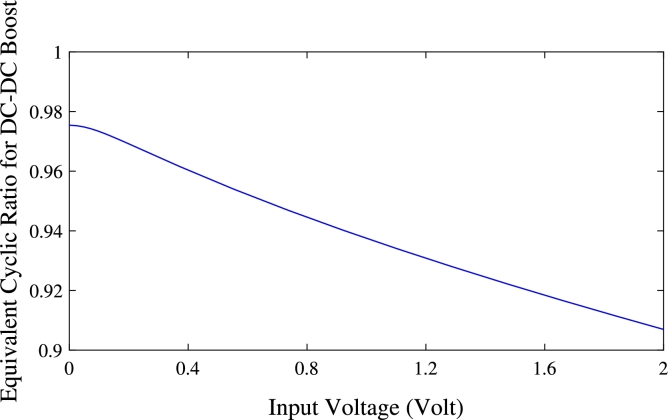
Duffing amplifier system cyclic ratio as a function of input voltage.

As a reminder Eq. (14) gave us the voltage amplification factor for a conventional boost chopper and Eq. (18) gave us the amplification factor for the proposed Duffing block. The two amplification factors are plotted in Fig. 5(a) where the solid line curve represents the amplification factor of the Duffing block and dashed lines, the amplification factor of the chopper. We can see from this figure that in the case of choppers, whatever the value of the cyclic ratio, the amplification factor is constant. So, for cyclic ratios lower than 0.9, the amplification factor is low and therefore not useful to have appreciable output voltages if we have very low input voltages. When considering the cyclic ratio of 0.9, it appears from the pink curve that we have a good amplification factor when using the Boost converter because it multiplies the input voltage by 10. Despite this interesting result, this type of amplification (through the chopper) cannot effectively help to amplify very low voltages such as those smaller or equal to 1*V*. By taking large values of the cyclic ratio such as 0.95 for example (black curve), the chopper will multiply the input voltages by a factor of about 20. This results to a good output voltage for low input voltages, but will also produce very large output voltages for large input voltages. Such a result is confirmed in Fig. 6(b), where it can be seen that for a duty cycle σ=0.95, the amplification factor is equal to 20, which corresponds to output voltages of 6*V* and 40*V* for input voltages of 0.3*V* and 2*V*, respectively. Therefore, for the converter to be used efficiently, another element should be designed that can detect the input voltage each time in order to regulate it according to the need. By analyzing blue curves of the amplification factor (Fig. 6(a)) and the output voltage (Fig. 6(b)) of the proposed system, the nonlinear aspect of the model leads us to different and interesting results. Curves in blue show very steep slopes in area (1), medium slopes in area (2) and low slopes in area (3). This simply means that with the proposed device, we have a high gain for low input voltage values, a medium gain for medium voltages and a low gain for high input voltages. This is due to the non-linear nature of the Duffing block that could be used effectively to amplify very low voltages from the PV. By drawing an analogy between the Duffing amplifier system and the DC-DC booster chopper system, we can determine the equivalent cyclic ratio $\sigma_{eq}$ of the Duffing amplifier system as:

(20) $$\sigma_{eq}=1-\frac{1}{g\left(x\right)}$$

A representation of this cyclic ratio as a function of the input voltage is given in Fig. 6. We can see that unlike the boost chopper's cyclic ratio which took constant values regardless the input voltage, the equivalent cyclic ratio of the Duffing amplifier system $\sigma_{eq}$ given by Eq. (20) depends on the input voltage *x* and decreases with increasing input voltage. This confirms once more the results of Fig. 6.

### 3.3 Experimental results

**Figure 7:**
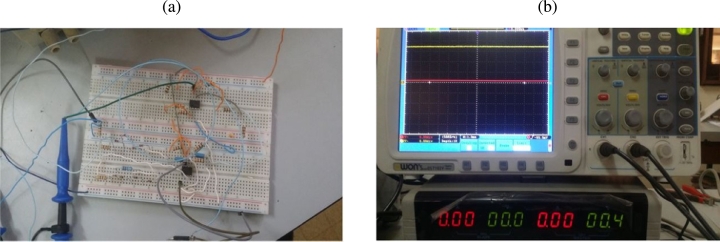
Experimental apparatus. (a): Proposed scheme model. (b): Voltage injection (stabilized power supply) and measurement equipment (oscilloscope).

In the previous section, all results were obtained theoretically. In this section, an experimental investigation is carried out in order to confirm the theoretical predictions. For this purpose, we realized the Duffing oscillator block as well as the summing-inverter block as shown in Fig. 7(a). In order to simulate the PV cell, we used a stabilized power supply, and visualized the results on a digital oscilloscope as shown in Fig. 7(b). The Duffing oscillator-based amplifier is implemented using 1N4148 as diode $D_{1}$-$D_{8}$, the TL082 (for its high slew rate, high input impedance, low supply current as low input bias current) as the OPAMP $U_{1}$, $U_{2}$, $U_{3}$ and $U_{4}$. The NLE resistors are set as $R_{1}=22k\Omega$, $R_{11}=R_{12}=4.7k\Omega$. The other parameters are set as in simulation. The OPAMP are supplied with ±15V using a DC-power supply. It is noticed that in practice, the difference between $y_{1}$ and $X_{1}$ (respectively $y_{2}$ and $X_{2}$) is less than $10^{-3}V$. Therefore, the two variables can be indifferently used.

**Figure 8:**
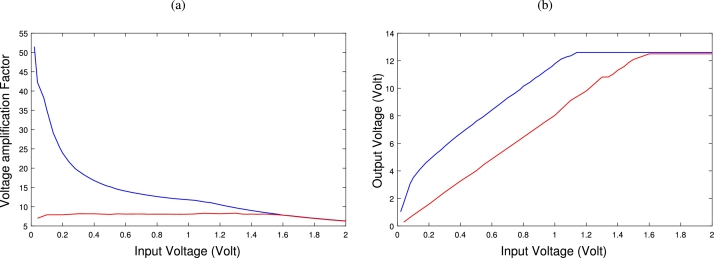
Experimental result. (a): Experimental plot of the amplification factor Vs/V as a function of V. (b): Experimental plot of the output voltage *V*_*s*_ versus the input voltage *V*.

From this experimental prototype, by varying the values of the input voltage, we obtain data on the output voltage and therefore the amplification. Fig. 8(a) shows the amplification coefficient and Fig. 8(b), the output voltage as a function of the input voltage. Blue line plots in both Fig. 8(a) and 8(b) represent the experimental results of the proposed system (Duffing non-linear oscillator) using 22kΩ as the main resistance of the non-linear element (NLE) and 4.7kΩ as the resistors to the diodes. Red curves are those of the linear oscillator using a standard resistor of 3.9kΩ (value representing the equivalent resistance of the NLE). It appears that the system does not only amplify in the presence of non-linearity but also self-regulates this amplification in favor of very small voltages as predicted by the theoretical results. Nevertheless, remarkable differences are noticeable. Firstly, for large values of the input voltage, we see the output voltage stabilizing at a maximum of 12.5*V*. The reason for this is that the output voltage of an OPAMP is always lower than its bias voltage, which has been set at 15*V*. Secondly, the amplitudes of the experimental curves are not in perfect agreement with those obtained in theory and this is explained by the fact that the value of p approximated in theory is different from the one used in practice but both the curves have the same shape reflecting the same phenomenon.

## 4 Conclusion

This work proposed and designed a device capable of amplifying very small voltages from solar cells when there is insufficient solar radiation. The device consists of a Duffing Oscillator, which amplifies and self-regulates the voltage generated by using its amplifier circuit and its nonlinear element. This oscillator is complemented by an inverter and a voltage adder to achieve the desired outcome. The Duffing oscillator amplifier is then coupled with a solar cell to quantify the voltage amplification ratio. Theoretical and practical studies were carried out and thanks to the results observed on the oscilloscope, we found that the theoretical and practical results agree. A comparative study between the proposed system and the conventional boost chopper was also conducted. However, in order to output sufficient power, we intend in prospect to include a current driver between the solar cell and the output of the U4 OPAMP. It was found that, due to its non-linear nature, the Duffing oscillator is a better amplifier of very low solar cell voltages with respect to the widely used boost chopper. It was also shown that the amplification factor of the proposed device varies according to the value of the voltage generated by the cell: it is high for low voltages and decreases with increasing input voltage, i.e. a sort of self-regulation of voltages by the device. Solar cell technology could therefore benefit greatly from the proposed device to solve the problem of low efficiency at low input voltages.

## Complete ethics statement

Review and/or approval by an ethics committee was not needed for this study because we are performing theoretical research and our experiments do not apply human or animals.

## CRediT authorship contribution statement

**Hermann Frederic Emakoua:** Writing – review & editing, Writing – original draft, Methodology, Investigation, Formal analysis, Data curation, Conceptualization. **Guy Valery Ayissi Eyebe:** Writing – review & editing, Writing – original draft, Formal analysis, Data curation, Conceptualization. **Annie Sylvie Wakata:** Writing – original draft, Project administration, Data curation, Conceptualization. **Jean Sire Armand Eyebe Fouda:** Writing – review & editing, Writing – original draft, Resources, Methodology, Investigation, Data curation, Conceptualization. **Martin Kom:** Conceptualization.

## Declaration of Competing Interest

The authors declare that they have no known competing financial interests or personal relationships that could have appeared to influence the work reported in this paper.

## Data Availability

No data was used for the research described in the article.
